# Book Reviews

**Published:** 1807-04

**Authors:** 


### CRITICAL ANALYSIS

### OF THE

### RECENT PUBLICATIONS

### ON THE

### DIFFERENT BRANCHES OF PHYSIC, SURGERY, AND MEDICAL PHILOSOPHY.

*Observations on the Effects of various Articles of Materia Medica, in the Cure of Lues Venerea: illustrated with Cases. The Second Edition, with Additions.* By John Pearson, F. R. S. Senior Surgeon of the Lock Hospital, and Asylum, and Surgeon of the Public Dispensary; Reader on the Principles and Practice of Surgery. 8vo. London, 1807.

The favourable reception of the former edition of this work, having so early called for the present, we need only take notice of those additions which the ingenious author has thought proper to make.

The

The Introduction has the following very important addition.

"12. It may be objected, indeed, against the foregoing observations, that although mistakes may have been sometimes committed, by not distinguishing the sequelæ of Syphilis, or the noxious effects of Mercury, from the specific action of the venereal virus, yet, many of the testimonies adduced in favour of these peculiar modes of cure, tend to demonstrate their efficacy in cases where no Mercury had been previously employed; and also, that the primary, as well as the secondary, symptoms of Lues Venerea have been cured by them.

"In proceeding to obviate what has been now alleged, I must, in the first place, express a doubt of the fact; not, indeed, whether sores on the penis, or tumours in the groin, have been cured without the aid of Mercury; but whether these symptoms, thus permanently removed, were in reality Venereal. What was the true nature of those complaints cannot now be ascertained: but if it be allowed, that appearances on the organs of generation, very much resembling the primary symptoms of Lues Venerea, do frequently take place from other causes; and if it be assumed as another fact, that Mercury is the only medicine yet known, that cures the venereal disease with certainty, we shall be assisted in forming a probable and not an incorrect opinion on the question before us. Every surgeon, who is engaged in much practice, must be frequently consulted on the nature of complaints, resembling the Chancre and the Bubo, which are not Venereal; and that which is not an unusual occurrence now, no doubt, presented itself as commonly in former times: for I think it will be difficult to assign a satisfactory reason, why an immunity from these morbid appearances should have been conferred on those who lived a century or two ago, and be refused to their posterity in the present day. The conclusion to be deduced from these remarks is obvious. Without imputing prejudice, perverseness, *mala fides*, or any other unworthy motive, to those writers who have published the narratives referred to, it will be quite sufficient to urge the imperfection of their history of Lues Venerea, as an apology for the incorrectness of their representations.

"It can scarcely be necessary to remind the reader, that the organs of generation, in both sexes, were frequently infested with very troublesome local diseases many ages before Syphilis was known in the world; and it cannot be presumed, with any colour of probability, that they were all finally extinguished on the appearance of that malady. Many of the diseases of these parts described by ancient writers, do certainly still occur; and I believe myself warranted to suspect, that new forms of disease not unfrequently arise, which are succeeded by a regular series of symptoms, nearly resembling the progress of Lues Venerea. An acquaintance with these and similar sources of error, must necessarily inspire a considerable degree of distrust, when we are presented with narratives, the leading circumstances of which are directly directly at variance with the best verified data in the history of Syphilis: nor will it imply either a want of deference, or of candour, to suppose the writer defective in the knowledge of his subject, rather than impute inconstancy and discordance to the order and method of nature.

"The spurious appearances to which I allude, are not, therefore, always to be regarded as the Sequelæ of Syphilis, or the effects of Mercury; since a distempered state of body, equal to the production of these morbid phenomena, may be the offspring of other causes, or a consequence of diseases, which have no affinity with Lues Venerea. I have not yet attained to that complete and satisfactory knowledge of the *Cachexia Syphiloidea*, which would authorize me to obtrude a publication on the subject; but the experience I have already had in the treatment of that multiform disease, has taught me, that it may appear under the following different circumstances.

"1. Where the Syphilitic virus has lately existed in the constitution, and the patient has employed the accustomed course of Mercury.

"2. Where the patient has been repeatedly diseased with Syphilis, and has used several courses of Mercury.

"3. Where a great length of time, from three to twelve, and sometimes twenty years, has elapsed since the patient has been exposed to the agency of the disease, and its remedy.

"4. After the Gonorrhœa, where small quantities of Mercury have been used.

"5. Where no venereal complaints, general nor local, have preceded the appearance of the *Cachexia Syphiloidea*; and where the patient has never been exposed to the hazard of contracting that disease, nor has laboured under complaints requiring the aid of Mercury.

"The three sources of error which I have now indicated; the sequelæ of Lues Venerea, the direct effects of Mercury, and the *Cachexia Syphiloidea*, may assist the student in solving many of the difficulties which will obtrude themselves, while he is studying the history and treatment of the Venereal disease. They may seem, at the same time, to acquit those of an unreasonable scepticism, who peruse the narratives of extraordinary cases, and marvellous cures, with suspicion and distrust. How much, or how little, our forefathers knew concerning these matters, would be an inquiry perhaps as unprofitable, as it would be unsatisfactory: since nothing could be more easy to an ingenious and well informed mind, than to adduce passages from early authors, in justification of any thing he may please to assert, and to infuse a sense and a meaning into detached expressions, far beyond what the writers themselves either taught, or conceived. Allowing every reasonable degree of merit to those original writers on Syphilis, who flourished before the 18th century, and much merit is undoubtedly due to many of them, yet it must be obvious to those who will take the trouble of examining their works, that their knowledge of some of the subjects to which I have now adverted, was too inadequate to command an implicit reliance either on their deductions, or their representations."

On this passage it is impossible not to make one remark. When a young practitioner starts his doubts, we are charmed with his modesty. But from one, who has appeared before the public nearly thirty years as an hospital surgeon, a public lecturer, and an author, we expect some information mixed with doubts. When we are told of varieties arising from so many causes and so complicated, of symptoms so easily confounded, yet requiring a mode of treatment directly opposite, and that from so experienced a guide we can at present expect no other assistance than the few hints contained in five short paragraphs—How dreary, how discouraging, must be the prospects of the student, or young practitioner! Must they not almost end in despair, when, on a re-examination of the assistance here held out, "while studying the history and symptoms of the venereal disease," he will be at a loss to know whether *Cachexia Syphiloidea* comprehends only the ulcer *mali moris* from a cachectic habit, or includes those local contagions, which Mr. Hunter first distinguished from the venereal, and which Dr. Adams afterwards traced back to Celsus.

When we make these remarks, it is far from our wish to detract from the merit of the work; and, perhaps, the error is with us in expecting too much.

In our further remarks, we shall only take notice of such passages as are either altered, or added, in the present edition.

At the close of the chapter on Sarsaparilla, the author very candidly corrects an error in his "Treatise on Cancer." remarking, that the disease there termed Elephantiasis should have been called *lupus* or *noli me tangere.*

In the 8th chapter on Ammonia Præparata, (page 102) is a note equally characteristic of the author's candour, and also another on the controversy between M. Peyrilhe and M. Fabre.

In the chapter on some effects of mercury, we have the following note.

"Although I am not possessed of any facts which confirm Mr. Hunter's opinion, yet I do not absolutely reject it: for every suggestion, offered by that extraordinary man, merits attention. I think I have, however, ample evidence, that free exposure to a cold and dry atmosphere counteracts the medicinal agency of Mercury, as certainly as this medicine resists the progress of Lues Venerea."

We do not conceive, that there is any material difference of opinion between Mr. Hunter and Mr. Pearson on this subject. Both admit that cold exasperates the disease and impedes the progress of the remedy. It is however probable, that Mr. Hunter may not have been sufficiently attentive to cautioning his patient against sudden exposure to cold air under severe courses of mercury; cury: if so, Mr. Pearson's remarks are not only very proper, but highly necessary.

The 13th chapter, on *Eczema mercuriale*, is altogether an addition to the present edition. It contains a much better account of what the author calls the mercurial rash, or, as it is called by some others, the Lepra mercurialis, than is to be met with in any other work.

Thesse, we believe, are the principal, if not the only alterations in the present edition. They are certainly sufficient to increase the value of a peformance, which was before well received by, and highly deserving the attention of, the medical world.

---

*A Treatise on the Varieties and Consequences of Ophthalmia; with a Preliminary Inquiry into its Contagious Nature.* By Arthur Edmonston, M. D. Fellow of the Royal College of Surgeons, and Honorary Member of the Royal Physical Society of Edinburgh. Edinburgh, 8vo. 1806.

In the year, 1802, Dr. Edmonston published a small Tract on the same subject, an account of which, the reader will find in page 185, vol. viii. of our Journal. The present work is very much enlarged. The author gives us an historical detail of the opinions of the various writers on the contagious nature of Ophthalmia, from Galen to the present times; after which, he goes at large into all the varieties of the disease; distinguishing their stages, causes, consequences, and methods of cure. Of these, we shall take notice in their order.

The "Preliminary Inquiry into the contagious nature of Ophthalmia," consists, first, of the historical sketch of the opinions entertained concerning the contagious nature of this disease. In this, the author shows, that till the late Egyptian Expedition, no clear reasoning on the contagious nature of this disease can be discovered, excepting in the Inaugural Dissertation of Dr. James Armstrong, *de tuenda nautarium sanitate;* printed at Edinburgh, 1789. On this occasion, Dr. E. laments, with much propriety, the number of valuable facts which are lost by the ephemeral existence of such productions. It must, however, be admitted, that when an author is anxious to extend an inquiry, he usually finds out these among other sources of information, and for such an event we are now probably indebted for the following valuable quotation from Dr. Armstrong's Thesis.

"Mense Januario 1782, regia navis Alba Marla, oram Hispaniolæ legens, navi servis onustæ obviam venit, unde tres nautas in se conscendere coegit. Unusquisque ex tribus, oculis leviter tum inflammatis laborabat, et, causam hujus affectus rogati, dicebant, se eo tempore ex dolentissimo convalescere morbo, quo omnes fere homines servifera in nave tum laborabant, et ne unum quidem præter dominum ipsum navis vim ejus effugisse. Quartum vero post diem quam in navem regiam allati fuerant, duo ex

 nautis nautis qui semper in hac nave fuissent, mane querebantur, sese proxima nocte acuto dolere in anteriore parte capitis correptos fuisse, et eodem tempore, molesto oculorum, haud secus ac si iis pulvis inspersus fuisset, sensu affectos. Postero mane, alii complures sese priore nocte correptos eodem modo et iisdem malis fuisse aiebant et septimi mane diei ex quo tempore primi duo affecti fuerant, viginti duo ad usitata munera præstanda ineptos hicce morbus jam reddiderat. Nonnulli propter dolorem capitis acutum, lecto affigebantur neque caput ex pulvino levare poterant, et inflammatio eò invaluerat, ut oculorum color carnem crudam quam maxime referret. Morbo tam cito in dies ingravescente, ne latius pateret, præfecto navis ægrotos omni cum sanis commercio interdicere necessarium visum est. Qua re facta, contagio non amplius viginti quinque affecit, et post quinque circiter hebdomadas quam primum, in navem advecta fuisset omnino evanuit."

Dr. E. remarks, that the only circumstances tending to weaken the support, given by this extract, to the contagious nature of ophthalmia, are the circumscribed scene of its operation, and the speedy and total destruction of this, supposed, contagious principle. In both these respects, it is said to differ from other instances of a similar natnre, hereafter to be stated.

Though we shall again meet our author on this subject, we cannot help stopping by the way to remark, that if the arrival of certain individuals from an infected ship, the consequent disease in a part of the crew, and the security of the remainder, by an immediate seclusion of the diseased from the sound, are not sufficient proofs of contagion; or if they do not confirm the reasoning drawn from " instances which are hereafter to be stated," we cannot help wishing, that our author had succinctly defined what he means by contagion. It is not enough, that, in the succeeding chapter, after giving us the history of the ophthalmia, as it appeared in the 2d Regiment of Argyleshire Fencibles, he enters regularly on the analogy between that and contagious diseases in general.

By the history, it appears, that the above regiment embarked in a healthy condition at Gibraltar, on board the Delft, which had brought troops from Egypt labouring under fever and ophthalmia. She was, however, well fumigated; the bedding was new, but the hammocks were the same as those occupied by the former crew. A lieutenant of the Delft, who had lost an eye in Egypt, and at that time laboured under the disease, was the only person on board who could not be considered in perfect health.

During the voyage from Gibraltar to England, one case of ophthalmia occurred twelve days after embarkation, and another four days after the first. A few slight cases of fever also occurred. The ship was crowded. The regiment remained ten days in Hilsea Barracks; and during that period twenty-one new cases occurred. The regiment was marched to Colchester, a distance of one one hundred and twenty miles, in eleven days. During the march four new cases occurred, all of them mild, and those before afflicted recovered.

Three days after arriving at Colchester the disease recurred with increased violence, and the invasion seemed very quickly to follow exposure to the cause. Seventy-five were infected when the regiment removed to Norman Cross. On the first and second day of the march the invalids complained of being worse; but during the remainder of the journey all of them mended. Twelve cases occurred on the road, all of which were slight.

The Middlesex militia were at Colchester, and seemed to have been infected by the Argyleshire. The officers of the latter escaped till the regiment was marched to Scotland. The Author gives the following account of the circumstances to which he attributes his own seizure.

"Medical men, from their hourly communication with every variety of disease, seem to acquire a kind of incapability of being affected by any; but if they intermit their professional labours for a time, they are equally liable with others. I happened to be absent from the regiment a few days when the disease was at its height. On my return, I was anxious to see the changes which the different cases had undergone, and was, perhaps, too minute in my examinations. That very same day I felt the sensation of a foreign body in the eye, the tunica adnata was inflamed, and a discharge of a watery fluid took place. But the assistant surgeon, who remained with the regiment during the whole of the time, escaped entirely."

Such is the history of this disease, of the appearance of which, Dr. E. remarks, that it had, in many instances, all the malignity of the Egyptian Ophthalmia. That it proved fatal to vision in no instance, he ascribes to early and repeated scarifications.

A few paragraphs follow, in which, the author finds no difficulty in showing, that the common causes of ophthalmia are insufficient to account for the progress of the above disease: After which, he attempts an "Analogy between the preceding ophthalmia, and contagious diseases in general."

"Some contagions," says our author, "such as lues venerea, require absolute contact to produce the effect, while in others, as small pox and measles, it is sufficient to breathe the atmosphere of the same room with the person under disease. But even in the most malignant cases, the sphere of action does not extend beyond a few feet from the source.

"If left to nature, they exhibit certain regular periods of rise and decline, and in these instances they seem to follow unequal periods. But if they are interrupted by art, these catenations of motions are broken, new associations are formed, and these changes cannot therefore be ascertained with sufficient accuracy.

"Contagion, like every other material substance, is susceptible of partial accumulation and diminution, and in general produces its effects according to the degree of concentration in which it exists. Every circumstance which prevents its free diffusion in the atmosphere, such as the crowded state of ships and jails, favours its accumulation and aids its operation.

"In most cases it seems necessary, in order to produce its full effects, that the body should be predisposed by debilitating powers, or, in other words, that causes which tend to change the state of its irritability had operated, although at times it extends itself under the most opposite circumstances.

"These are a few of the leading laws of all contagious diseases; and the Ophthalmia which occurred in the second regiment of Argyleshire Fencibles, exhibits, in its origin and progress, a striking coincidence with them.

"Thus, a certain period elapsed before it made its appearance, and it occurred chiefly among the soldiers, whose confined situation, and the impure atmosphere which they breathed, materially affected the irritability of their systems, rendering them more easily affected by any noxious power; but when its energy became increased, it extended itself in every direction.

"Sleeping in the same room, or approaching near to the eye of a person labouring under the disease, was sufficient to produce it in another person. In this way almost all contagions operate.

"The evening exacerbation and morning remission partake of the general nature of febrile disease; and the head-ach, restlessness, white tongue, and irregularity in the state of the circulation, are proofs that it existed.

The third day was usually the period of change. Contagious diseases follow unequal periods. But in this case, as recourse was had early to medical assistance, the different gradations became less distinctly observable.

"The march from Portsmouth to Colchester, in some measure arrested, although it could not destroy, the influence of the cause producing the Ophthalmia. Variety of scenery, and changes of situation, produce similar effects upon the hooping cough, small-pox, and other contagious disorders.

"The march to Norman Cross had a similar effect, but as the disease had existed for a longer period, and individual cases were becoming worse, exposure to exciting causes naturally aggravated the symptoms. The same takes place, and must necessarily do so, in every disease where particular organs are chiefly affected.

As the author considers these to be "the few leading laws of all contagious diseases," we might suppose, he intends we should understand, that in these few, all such diseases agree. But in the first paragraph he has marked an essential difference. This is, however, of less consequence than he seems to make it, because contact, whether by the diffusion of matter in the air, or by two palpable substances, is still contact. But where is the analogy between the venereal disease, and one which if left to nature, has its periods of rise and decline? or what interruption of art can break break the CATENATION OF MOTIONS, or form NEW ASSOCIATIONS in small-pox or measles, so as to prevent our "ascertaining the changes with sufficient accuracy?"

"Again, what is the partial accumulation and diminution, according to the degree of concentration, that alters the effects of small-pox and measles? That the crowded state of ships and jails "favours accumulation and aids the operation," we are ready to admit, if these expressions are confined to hospital and jail fever, in which the effect on those exposed is more powerful, in proportion as the above causes are increased. But nothing of this kind is found in the contagion of small-pox and measles. In these, provided the impregnation of the air is sufficient to excite the diseased action, we have no authority to say, that such diseased action, in the person infected, bears any ratio to the degree of impregnation in the air.

The author next adduces "proofs, that the preceding ophthalmia was occasioned by the operation of a specific contagion." In this we find allusion to the "analogy" we before objected.

A statement of facts follows, quite sufficient to convince us that the disease was communicated from one individual to another; but we wish, on many accounts, that the remainder of this chapter had been omitted. The discussion on contagion, which follows, is more diffuse than the former, and replete with intended illustrations, which render the subject more obscure than ever.

Our author next relates the account of an ophthalmia, as it appeared in Paris and its environs in 1803, of which he was a witness. He takes some pains to prove that this disease could not be a mere epidemic from some unknown properties in the atmosphere, but must have been propagated by contagion. But he is by no means so satisfactory in his proofs on this occasion, as in his account of the ophthalmia in the Argyleshire Fencibles, For though it is certain that the disease appeared at different times in Paris, St. Cloud, and Versailles, and even under different states of the atmosphere, yet we know that epidemics travel in this manner, and we are by no means sufficiently acquainted with the properties of the atmosphere by which they are influenced. The author shows, indeed, that epidemic fevers are influenced by changes of weather; but these kind of fevers seem peculiar to climates south of the meridian of Paris. This part of the work concludes with an enumeration of "the objections to the opinion which considers ophthalmia as a contagious disease." All which are candidly answered.

Had Dr. Edmonston concluded his book here, he would, in our opinion, have made it longer than necessary; indeed, we have met with very little which might not have been added as an Appendix to his "Account of Ophthalmia," published in 1802. But we confess our patience is put to a most severe trial, when we find, tacked at the end, more than 300 pages on the History of Ophthalmia, its varieties, causes, consequences, and treatment. If we had met with a single satisfactory remark, that is not contained in

Professor

Professor Scarpa, and others, who have recently come before us, we would gladly have offered it to our readers. We mean not to say, that Dr. Edmonston has copied altogether from that useful work, any thing more than the arrangement; but where he differs from the Professor, our judgment has always been in favour of the latter. We shall not be expected, after this, to enter largely on this part of the work; but to satisfy our readers that we are not actuated by partiality, or disposed to misrepresent, we shall confine our remarks to a single *Section*, least connected with the general subject.

Symptomatic Ophthalmia is thus introduced.

"Although various diseases give a predisposition to Ophthalmia, yet there are only two which can be said to modify its symptoms, and on many occasions to determine its duration. These are *scrophula* and *lues venerea*."

From such an introduction our readers will expect equal obscurity to continue throughout; and they will not be disappointed. Scrophula is a word of such general use, and so rarely defined, that, in our opinion, it is no better in the author's hands, than lentor of fluids, (which he begins with condemning) in the hands of his predecessors. After a few words on the scrophulous "*Diathesis*, or temperament," which is said to be marked by a general flacidity and mobility of fibre" (a most perspicuous definition!) we have an account of the manner in which Ophthalmia is modified by such a predisposition. The description might be much better referred to Professor Scarpas's chronic inflammation. But there is something peculiarly loose in the introductory part of the Venereal Ophthalmia; "like scrophula, says our author, the syphilitic virus, when diffused through the system, frequently affects the eyes, and induces a particular species of Ophthalmia." By this are we to understand, that Scrophula is a *virus*, which we were just now told was a *diathesis?* The description which follows is principally from Mr. Bell, including one symptom, which he remarks "has seldom been observed, and which is apt to be mistaken for an incipient fistula lachrymalis."

But what principally engages our author's attention, is the Ophthalmia which arises from a suppressed gonorrhœa. After considering the various opinions on the subject, it must be admitted, that he falls into the most probable, viz. that if the Ophthalmia arises immediately on the cessation of gonorrhœa, it should be ascribed to sympathy, and not to a metastasis of matter. A metastasis of violent inflammation is by no means uncommon, but our experiences does not justify the occurrence of such an event from the urethra, in virulent gonorrhœa, to any other part than the testicle; and we are glad to be supported in this opinion by such men as Mr. Ware and Mr. Pearson, each of whom has as large opportunities of deciding such a question, as the metropolis can afford. Fortunately, however, the question is not very important, for if only the inflammation is transferred without without the virus, the disease must be treated according to the symptoms which appear, without regarding the cause.

---

*An Essay on* Ophthalmia; *containing a History of that Disease, as it appeared in the First Battalion of the* 89*th Regiment, with some Observations on its Causes and Symptoms. Also the Medical Treatment, &c. which have been crowned with unparalleled Success. In a Letter to* James M'Gregor, m. d. *Deputy Inspector of Army Hospitals.* By Henry Reid, Surgeon of the First Battalion, 89th Regiment, and Member of the Physico-Chirurgical Society, Dublin.

The progress of this disease is traced with some accuracy; but we very much regret that "delicacy," which will not permit the author to explain the causes of so sudden an increase in the numbers affected, which is stated to have been from sixteen to seventy in one month.

The author considers, as the most alarming appearance, a violent tumefaction of the upper eye-lid, which is discoloured by a blueish tinge, accompanied with fever and shooting pains. The eye is closed, in consequence of which the increased secretion, if not occasionally dislodged, will ulcerate through the cornea, suffer the humours to escape, and the eye will be irrecoverably lost. This great tumefaction is ascribed to the inflammation of the lachrymal gland.

The inquiry next commences, whether the disease is infectious, which is determined in the affirmative, though many difficulties are admitted to attend a complete proof. In a note, it is said, Mr. Marshall has put the question to experiment, by applying some matter to his own eye, which produced considerable smarting pain for the space of an hour, but the disease did not establish itself. He communicated the infection to three other men, two of whom had the regular disease. If all four had taken it, there would have been a fair presumption that contagion was the cause. But when we consider that these men were obnoxious to the common cause, without the immediate application of the matter, we should not be satisfied with the result of this experiment, though it is not our intention to question the contagious property of the disease.

The treatment which our author found so eminently successful, was early and copious bleeding, according to the symptoms. The topical applications were warm poultices of bread and milk. He recommends as early a separation as possible for the convalescents, and every attention to the general health, as well as the local symptoms.

This letter is extremely defective in style, and sometimes in accuracy; but these are of little consequence, compared with the important facts it contains.

*Observations*

*Observations on the Humulus Lupulus of Linnæus; with an Account of its Use in Gout, and other Diseases: with Cases and Communications.* By A. Freake, Apothecary, Tottenham Court Road. Second Edition, 8vo. 1807.

We have already taken notice of the first edition of this Pamphlet, which, as the writer remarks, was circulated, principally, among the faculty, that the value of the remedy might be with more certainty estimated. The trial has answered every expectation the author could wish. Four Communications are annexed, from four Fellows of the London College. Drs. Latham and Mayo, authorize Mr. Freake to use their names; and Drs. Stone and Maton, add their signatures to Letters confirming the value of the remedy. Dr. Stone does not indeed consider the hop in a much higher view, than as an useful variety with the other bitters; which, however, he conceives, it may supersede with advantage, not only on account of its more agreeable taste, but for its aperient qualities. Dr. Maton writes much more at large on the subject; after whose letter we have the following

"Concluding Observations.

"I have now, for nearly six years past, administered the Lupulus in a variety of diseases, and I can with confidence assert that it is a very valuable medicine. I have not preserved an account of all the trials I have made of it, but I think I should be correct in asserting, that it has afforded relief in more than one half of the cases in which I have given it. I do not recollect a single instance in which I have had occasion to regret the employment of it; for, although a slight giddiness in the head has occasionally been produced, its duration was always very short. Dr. De Roche seems apprehensive that the saturated tincture may occasion diarrhœa and pains in the bowels. I have not observed either these or any other unpleasant effects from it, nor has its administration prevented the use of other medicines as auxiliaries."

---

*Practical Observations on Urinary Gravel and Stone; on Diseases of the Bladder and Prostate Gland; and on Strictures of the Urethra.* By Henry Johnson, Fellow of the Royal College of Surgeons, at Edinburgh. Edinburgh, 8vo. 1806.

It is not often that we meet with a book so completely unexceptionable as the present, and, we are obliged to add, from which we have learned so little. It is certainly a compendium of most that has been written on the subjects mentioned in the title page; nor is the author backward in offering his own opinions. But the latter are too often trite, as well as modest, and, in a few instances, sufficient credit is not given to the authors quoted. The latter, is, however, rarely the case. We shall only mention Mr. Whateley's improvement in substituing kali purum for lunar caustic, stic, in the treatment of strictures in the urethra. The author does not seem aware, that the principal advantage proposed by the kali purum, is, that by its property of combining and forming soap with animal matter, a more complete solution of the stricture is procured, and the necessity afterwards of casting off a slough is avoided. It is not our intention to enter into the inquiry, which mode of operating may be the best, but only of showing, that Mr. Johnson has not sufficiently explained the comparative properties of the two.

